# Degradable allyl *Antheraea pernyi* silk fibroin thermoresponsive hydrogels to support cell adhesion and growth

**DOI:** 10.1039/d1ra04436b

**Published:** 2021-08-23

**Authors:** Boxiang Wang, Hangdan Xu, Jia Li, Dehong Cheng, Yanhua Lu, Li Liu

**Affiliations:** School of Materials Science and Engineering, Shanghai University Shanghai 200444 China liuli@staff.shu.edu.cn; Key Laboratory of Functional Textile Materials, Eastern Liaoning University Dandong 118003 Liaoning Province China yanhualu@aliyun.com

## Abstract

At present, *Antheraea pernyi* silk fibroin (ASF) based hydrogels have wide potential applications as biomaterials because of their superior cytocompatibility. Herein, ASF is used as a nucleophilic reagent, reacted with allyl glycidyl ether (AGE) for the preparation of allyl silk fibroin (ASF-AGE). The investigation of ASF-AGE structure by ^1^H NMR and FTIR are revealed that reactive allyl groups were obtained on ASF by nucleophilic substitution. A series of ASF based hydrogels are manufactured by *N*-isopropylacrylamide (NIPAAm) copolymerization bridged with ASF-AGE. By the silk fibroin self-assembly process, stably physical cross-linked hydrogels are formed without any crosslinking agent. These hydrogels exhibit good thermoresponsive and degradability, for which the LCST was about 32 °C, and these hydrogels can be degraded in protease XIV solution. Excellent cell proliferation, viability and morphology is demonstrated for b End.3 cells on the hydrogels by the characteristic MTT assay, CLSM and SEM. The cytocompatibility of b End.3 cells was demonstrated with excellent cell adhesion and growth on these ASF based hydrogels *in vitro*. These degradable and thermoresponsive ASF based hydrogels may find potential applications for cells delivery devices and tissue engineering.

## Abstract 2

At present, *Antheraea pernyi* silk fibroin (ASF) based hydrogels have wide potential applications as biomaterials because of its superior cytocompatibility.

## Graphical abstract

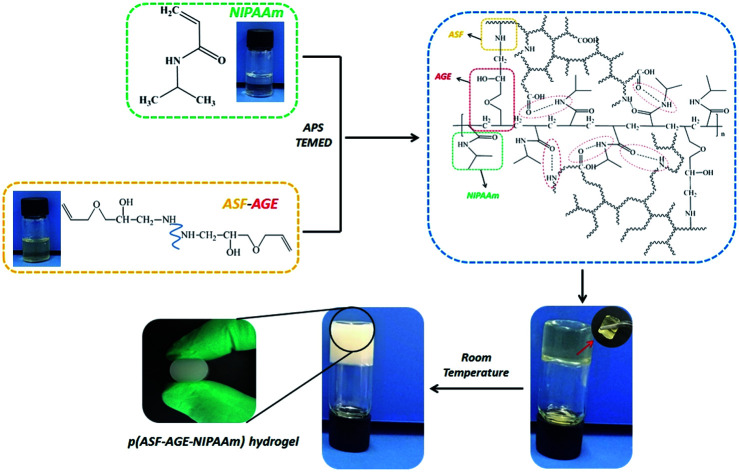

## 1. Introduction

Protein-based hydrogels have been recently pursued as important materials for biological scaffolds in tissue engineering,^1^ cell culture,^2^ and artificial cartilage^3,4^ because of natural structural proteins displaying critical structural and bioactive properties that have evolved in nature for millions of years.^5^ Most of these hydrogels are highly similar to the native extracellular matrix in terms of bioactivity, and unique degradation and flexible mechanical properties provide the opportunity to achieve various biological functions that are beneficial for cell engineering. A number of naturally protein and synthetic polymers have been studied as building blocks for hydrogel formation due to their good biocompatibility and biodegradability.^6–8^ Recently, blending (mixing) proteins with synthetic polymers is a technological approach to generate protein based hydrogels with more complete set of specific properties. Additionally, many crosslinking approaches have been utilized to fabricate covalently cross-linked networks of hydrogels.^9^ However, chemical crosslinking systems aldehydes, free radicals or small molecule chemical crosslinking agents may cause potential toxicity towards the encapsulated cells.^10^ Hence, generating multifunctional, non-toxic and biodegradable hydrogel biomaterials based on structural protein is emerging as a useful direction in the field to specific medical needs *in vitro* and *in vivo* at present.

Silk fibers are one of nature's most highly engineered materials with excellent characteristics, such as high tensile strength, Yong's modulus, toughness, extensibility and superior to synthetic fibers. Silk proteins (fibroin and sericin) is the main component of silk which is produced by silkworms are broadly classified into two types, namely mulberry (*Bombyx mori*) and non-mulberry.^11^ Silk sericin (SS) is an inexpensive glycoprotein obtained as a by-product in the silk industry which is mainly consists of random coils forming an amorphous phase and high polarity of SS enables easy cross-linking and blending with other polymers to obtain biomaterials with improved properties. SS has been widely applied in tissue engineering because of its variable amino acid composition and diverse functional groups confer upon it attractive bioactive properties.^12^ Silk fibroin (SF) is a natural fibrous protein with favourable capability of enhancing attachment that has been widely used as a matrix for tissue regeneration of bone, cartilage, skin, blood vessels, nerve and other tissues. SF based scaffold can mimic the extracellular matrix that fabricated into various kinds of constructs for proliferation and differentiation of chondrocytes.^13^ Y. Cheng *et al.* reported a SF hydrogel reinforced by short silica nanoparticles with a superior osteoinductive property for treating bone defects and the study confirms that the reinforced hydrogel with mechanical properties and osteoinduction enhancement facilitate bone repair.^14^ Furthermore, various forms of SF or SF-based composite biomaterials are fabricated by using diverse methods such as freezing-drying, salt leaching, gas foaming, electrospinning and hydrogels.^13^ G. Cheng *et al.* summarized the recent advancements of SF in cartilage regeneration and the fabrication methods of SF based scaffold in cartilage tissue engineering, the sterilization methods used for the silk fibroin-based scaffolds were also discussed in detail.^13^ SF is often used with other biomaterials such as chitosan, collagen, poly l-lactic-acid, agarose, hyaluronic acid and cellulose as scaffolds for tissue engineering for the study.^13^ J. Chen *et al.* reported a cardiac patch that fabricated with electrospinning cellulose nanofibers modified with chitosan/silk fibroin (CS/SF) multilayers *via* layer-by-layer (LBL) coating technology and the research suggests that the CS/SF nanofibrous cardiac patch loaded with AD-MSCs could be an effective and recommended strategy for stem-cell-based MI therapy.^15^ Hence, SF comes to the fore in all realms except the dress field because of its excellent and unique properties, such as nontoxicity, tunable mechanical properties, biodegradability, acceptable biocompatibility and the favourable capability of enhancing attachment.

The wild non-mulberry silkworm species are heterogeneous and have a wide distribution throughout the world in particular.^16^ China is one of the largest producers of commercial non-mulberry silks (*Antheraea pernyi*). *A. pernyi* is a wild non-mulberry silkworm species belonging to the Saturniidae family, which is commonly known as Chinese temperate (oak) tussah. *A. pernyi* silk fibroin (ASF) is composed of alanine (43.07%), glycine (27.27%), serine (11.26%), tyrosine (5.26%) and aspartic acid (4.47%), in opposition of *Bombyx mori* silk fibroin, which consists of highly repetitive glycine–alanine–glycine–alanine–glycine–serine (GAGAGS) sequences. ASF contains inherent arginyl-glycy-aspartic acid (RGD) peptide sequences, distinguishing it from other silk fibroins, which is binding sites of cell integrin receptors. Compared to *Bombyx mori* silk fibroin, RGD sequences in ASF mediate the interactions between mammalian cells and extracellular matrices which facilitate improved cell adhesion and proliferation.^17,18^ Therefore, ASF based biomaterials have attracted research efforts to investigate it as materials for fabrication of biomedical devices, including tissue engineering, cell culture substrates, bone regeneration, bioactive controlled release carriers and gene delivery systems, due to its superior cytocompatibility. For instance, it has been reported that *A. pernyi* silk fibroin scaffolds promote the adhesion and proliferation of the tenocytes *in vitro*, and repaired the defect of tendon in rabbit model.^19^ At 16 weeks after implantation *in vivo*, the surface of the neo-tendon was smooth and the bundle of collagen fiber was uniform and well arranged. However, the authors also found that only the external part of the ASF braided scaffolds degrade at 16 weeks after implantation, suggesting poor degradability. Therefore, the degradation pattern of ASF based scaffolds need to be appreciated in future research.

In order to minimize potential cytotoxicity, controlling the self-assembly of silk fibroin was one way to produce ASF based hydrogels without any crosslink agent, resulting in the further improvement of cell interactions. It has been reported that a mild self-assembly process to prepare porous and nanofibrous silk-based scaffolds from aqueous solution.^20^ The nanofibrous architecture of self-assembly silk-collagen scaffolds was similar to natural ECM, provided fibroblasts with a favorable microenvironment to enhance growth and proliferation. Meanwhile, use of affinity techniques in protein modified has become a routine method in biochemical research.^21,22^ Silk fibroin can be chemical modified and cross-linked with an epoxy group by its reactive groups of amino, hydroxyl or carboxyl.^23^ It has been found that epoxide was used to produce silk fibroin based medical material.^24^ In previous studies, epoxidized modification of silk fibers to improve the tenacity, moisture regain, water retention capacity and dyeing property has been widely research.^25^

Herein, we demonstrate the preparation of ASF based hydrogels with good thermoresponsive and degradability, which can support cell adhesion and growth. ASF was used as a nucleophilic reagent, which reacted with allyl glycidyl ether (AGE) for the preparation of allyl silk fibroin (ASF-AGE). The AGE not only reacted with ASF, but also bridged poly *N*-isopropyl acrylamide (PNIPAAm) to form ASF based hydrogel network by copolymerization. The structure of ASF-AGE was researched by ^1^H NMR and FTIR. The obtained p(ASF-AGE-NIPAAm) hydrogels showed good thermoresponsive and degradability, which the LCST was about 32 °C, and these hydrogels can be degraded in protease XIV solution. The cytocompatibility of the ASF based hydrogels were investigated by evaluating the ability of the hydrogel to support cell adhesion and growth *in vitro*.

## 2. Experimental

### 2.1 Materials

Raw silk cocoons of *Antheraea pernyi* (Liaoning Tussah Silk Institute Co., Ltd, Dandong, China) were used for preparation of ASF. Ally glycidyl ether (AGE) and protease XIV were purchased from Sigma-Aldrich. *N*-Isopropylacrylamide (NIPAAm) (98%, Aladdin Bio-Chem Technology Co., Ltd, Shanghai, China) was purified by dissolution and recrystallization in hexane. All other materials and reagents were purchased from Sinopharm Chemical Reagent Co., Ltd, and used as supplied.

### 2.2 Preparation of regenerated *A. pernyi* silk fibroin (ASF)

**Fig. 1:**
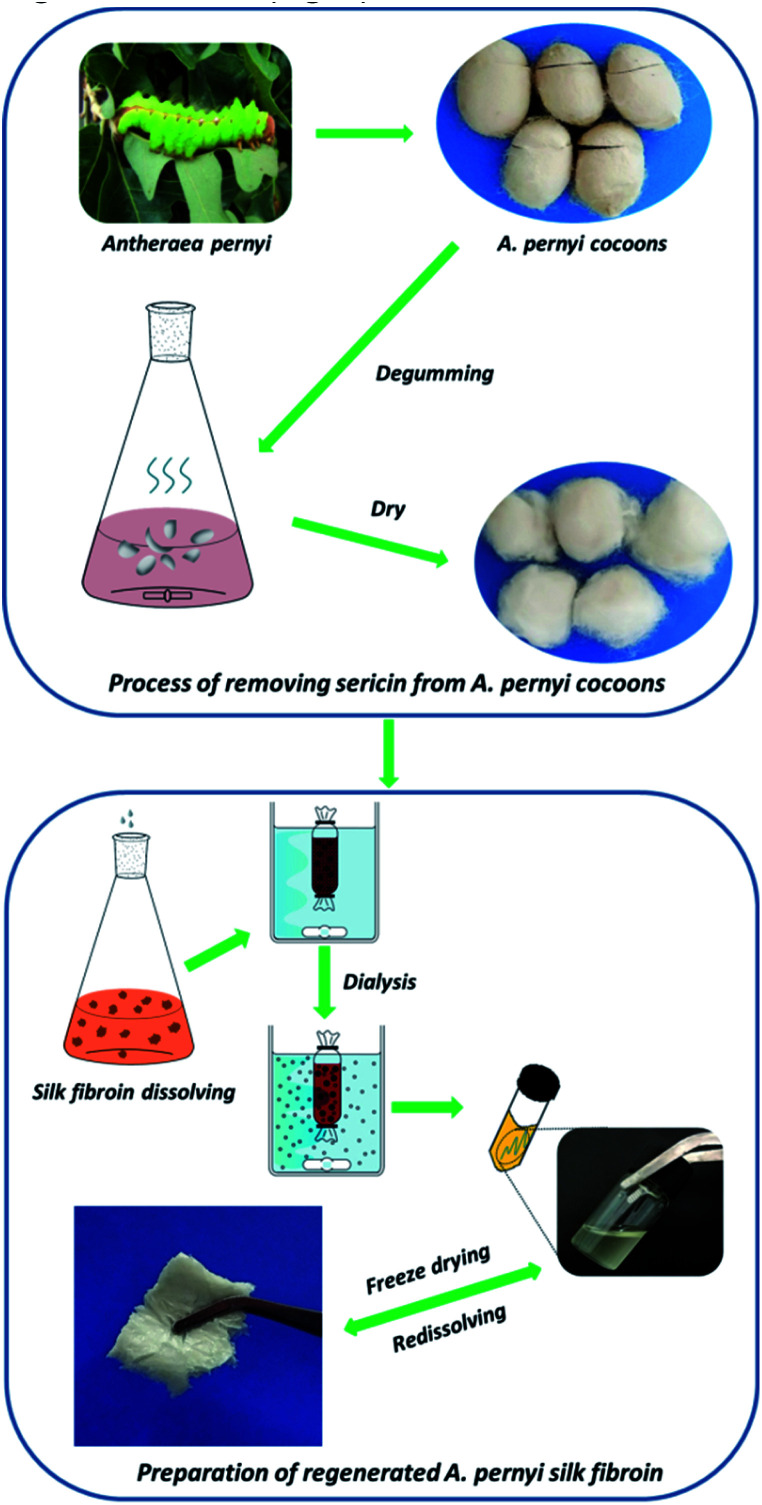
Preparation of regenerated *A. pernyi* silk fibroin.

Regenerated ASF was extracted following the procedure as per earlier established literature. Briefly, raw cocoons of *A. pernyi* were degummed by boiling three times in 0.25% (w/v) Na_2_CO_3_ solution at 100 ± 2 °C or 30 min to extract the traces of sericin, rinsed thoroughly with distilled water, and dried in an oven. After drying, the degummed fibers were dissolved in molten Ca(NO_3_)_2_ at 105 ± 2 °C for 4 h. The rough yellow regenerated ASF solution was then filtered. The mixed solution was dialyzed against deionized water for 3 days with an 8–14 kDa molecular weight cut off dialysis tube to remove salts. The solution was finally centrifuged at 8000 rpm for 10 min to remove impurities. The pure regenerated ASF solution was frozen at −50 °C for 12 h, followed by freeze-drying for 48 h to obtain regenerated ASF (Fig. 1).

### 2.3 Preparation of allyl silk fibroin (ASF-AGE)

A dried and weighed regenerated ASF sample was padded into 250 mL three-necked round-bottomed flask with 50 mL distilled water dissolved. The ASF solution was treated by 2 M Na_2_CO_3_ and 1 M NaOH solution to adjust pH. Then nitrogen was continuously injected into flask together with allyl glycidyl ether drip into it slowly. This flask was joined with a reflux condenser, reacted in a thermostatically controlled bath with stirring at the desired temperature. At the end of reaction the mix solution was treated by 1 M HCl solution to adjust pH to neutral. After dialyzing against deionized water at 4 °C for 3 days, the solution was rapidly frozen at −50 °C for 8 h, then allyl silk fibroin was obtained by freeze-dried.

### 2.4 Formation of p(ASF-AGE-NIPAAm) hydrogel

NIPAAm was dissolved in a mixture of toluene and *n*-hexane (6 : 4, v/v) solution and a concentration of 15 wt% of NIPAAm solution were prepared. The NIPAAm solution was heated to 60 °C in a thermostatically controlled bath and cooled at room temperature, then placed in a −5 °C fridge until crystal was completely precipitated and the purified NIPAAm was obtained. ASF-AGE was dissolved in deionized water to a concentration of 5% (w/v), then mixing with APS and TEMED in an ice bath. NIPAAm (75 mg mL^−1^) solution was slowly dripped into the mixed solution with nitrogen injected continuously at 20 °C. After a period of reaction the stickiness solution was obtained, the solution was injected into a glass mold and nitrogen was bubbled into the solution for 30 min. In the end, the glass mold was quickly covered with a Teflon stopper. This solution was polymerized by free radical reaction after incubating at room temperature for 12 h. After reaction, the hydrogels were dialyzed against deionized water for 2 days with an 8–14 kDa molecular weight cut off dialysis tube to remove unreacted chemicals. The formulations for the synthesis of p(ASF-AGE-NIPAAm) hydrogels were listed in Table 1.

**Table 1:** The formulation for the synthesis of p(ASF-AGE-NIPAAm) hydrogels

Hydrogel code	ASF-AGE : NIPAAm (mass ratio)	ASF-AGE (mg)	NIPAAm (mL)	APS (mg)	5% TEMED (μL)
pAGN-1	1 : 9	50.0	6.0	9.5	60
pAGN-2	1 : 4	100.0	5.33	7.9	60
pAGN-3	1 : 2.5	142.9	4.76	7.1	60
pAGN-4	1 : 1.5	200	4.0	6.0	60
pAGN-5	1 : 0.75	285.7	2.86	4.3	60

### 2.5 Structure analysis of ASF-AGE

#### 2.5.1 ^1^H nuclear magnetic resonance (^1^H-NMR)

50 mg products of ASF and ASF-AGE were dissolved in 1 mL of deuterium oxide. The ^1^H-NMR spectra of each sample was recorded on a Bruker AV400 NMR spectrometer (Bruker, Germany), with an operating frequency at 400 MHz.

#### 2.5.2 Fourier transform infrared spectroscopy (FTIR)

The structure of ASF-AGE was also analyzed by FT-IR (Nicolet IS10 FT-IR spectrometer, Thermo Fisher Scientific, USA). The pure sample solids were prepared in KBr (Sigma-Aldrich) pellets for FTIR spectra analysis. All infrared spectra were recorded in the range of 4000–500 cm^−1^ using an accumulation of 32 scans with a resolution of 4 cm^−1^.

### 2.6 EMC and LCST measurement of hydrogels

The equilibrium moisture content (EMC) was defined as the weight of absorbed water per weight of the dried hydrogel. The EMC of hydrogel samples were gravimetrically monitored by immersing the dried hydrogel samples (*W*_d_) in excessive PBS solution in the range from 15–50 °C. The mass of swollen hydrogels (*W*_s_) was measured after removing the surface water. The experimental results were calculated from an average of three samples and the formula was EMC = (*W*_s_ − *W*_d_)/*W*_s_ × 100%. The LCST was defined as the temperature at the highest point of the derivation curve of each temperature EMC absolute value *vs.* temperature to research the thermoresponsive of the hydrogels.

### 2.7 Degradation rate measurement of hydrogels

The freeze-dried hydrogels were cut 1 ± 0.5 cm × 1 ± 0.5 cm × 1 ± 0.5 cm block, and incubated in 5.0 U mL^−1^ protease XIV PBS solution (bath ratio 1 : 80) for 1–28 days at 37 °C under slow shaking. Degradation rate was expressed as the percentage of weight loss relative to the initial dry weight.

### 2.8 Cell biocompatibility

Brain microvascular endothelial cells (b End.3) (frozen in −80 °C ultra-cold storage freezer) were cultured in 4 mL Dulbecco's Modified Eagles Medium (DMEM, Meilun-bio, China) which was mixed with 10% fetal bovine serum (FBS, HyClone, USA), 100 U mL^−1^ penicillin and 100 μg mL^−1^ streptomycin at 37 °C in an atmosphere of 5% CO_2_ in air. In the logarithmic growth phase, the cells were trypsinized using 0.25% trypsin (Invitrogen, USA) and resuspended at 1.0 × 10^5^ cells per mL in fresh DMEM with FBS and antibiotics. A 100 μL aliquot of cells suspension was inoculated in each well of 96-well cell culture plates to cover the p(ASF-AGE-NIPAAm) hydrogel samples (*n* = 6), 100 μL aliquot of cells without samples were control group, and blank group was not inoculated with cells. The 96-well cell culture plates were kept in a humidified atmosphere of 95% air and 5% CO_2_ at 37 °C for 1, 3, 5 and 7 days. After culturing, a part of cells were washed three times with DMEM. The medium was replaced with 100 μL DMEM mixed with 10 μL MTT agent for 4 h for the cell viability test. After incubation, the reaction was carefully removed from each well, and 100 μL dimethylsulfoxide (DMSO) was added. The plates were gently agitated until the blue-purple crystal (formazan precipitate) completely dissolved, and the absorbance at 490 nm was measured using a Synergy H1 microplate reader (Bio-Tek, USA). The other cells were dipped into 4% paraformaldehyde solution for 15 min, and washed with PBS solution three times. These cell samples were blocked with PBS containing 1% Triton X-100 by staining with DAPI (Sigma, USA). After incubated in dark for 40 min at room temperature, a confocal laser scanning microscope (CLSM) (FV-1200, Olympus, Japan) was used to observe the cells adhering to the sample.

### 2.9 Scanning electron microscope (SEM) observation

After 1, 3, 5, 7 days incubation with b End.3 cells on the hydrogel, the hydrogel samples were washed three times with PBS and fixed in 4% formaldehyde for 15 min, then washed further three times with PBS. The freeze-dried hydrogel samples were coated with gold and observed by SEM (JSM-IT100, JEOL, Japan).

## 3. Results and discussion

### 3.1 Synthesis of allyl silk fibroin

**Fig. 2:**
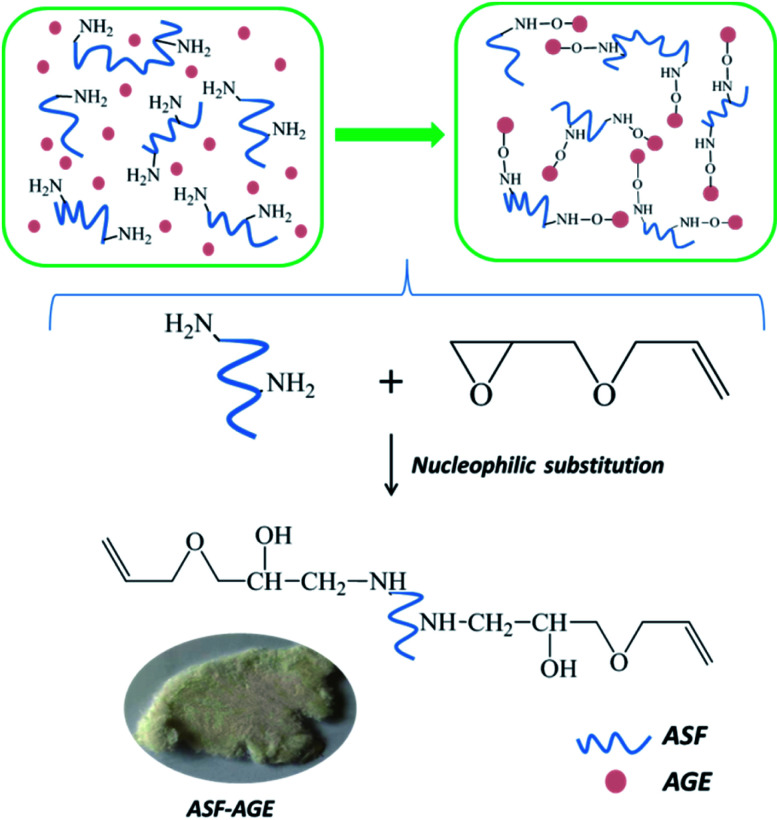
A schematic illustration of the synthesis of ASF-AGE.

The freeze-dried ASF was dissolved in deionized water, and the ASF solution was adjusted to 10 mg mL^−1^. The pH of ASF solution was adjusted from 9 to 11 with a NaOH and Na_2_CO_3_ solution. In this condition, the AGE solution was gradually dripped into it, nucleophilic substitution was occurred with amino group in ASF and epoxy group in AGE. The aggregated structure of ASF in solution shows random coil structure, and numerous terminal amino groups are exposed. At pH > 8.0, a nucleophilic substitution reaction was occurred by amine groups of ASF with epoxide groups of the diglycidyl ether (Fig. 2). Base-catalyzed ring opening of the epoxide groups occurs predominantly at the least hindered carbon atom.^26^

**Fig. 3:**
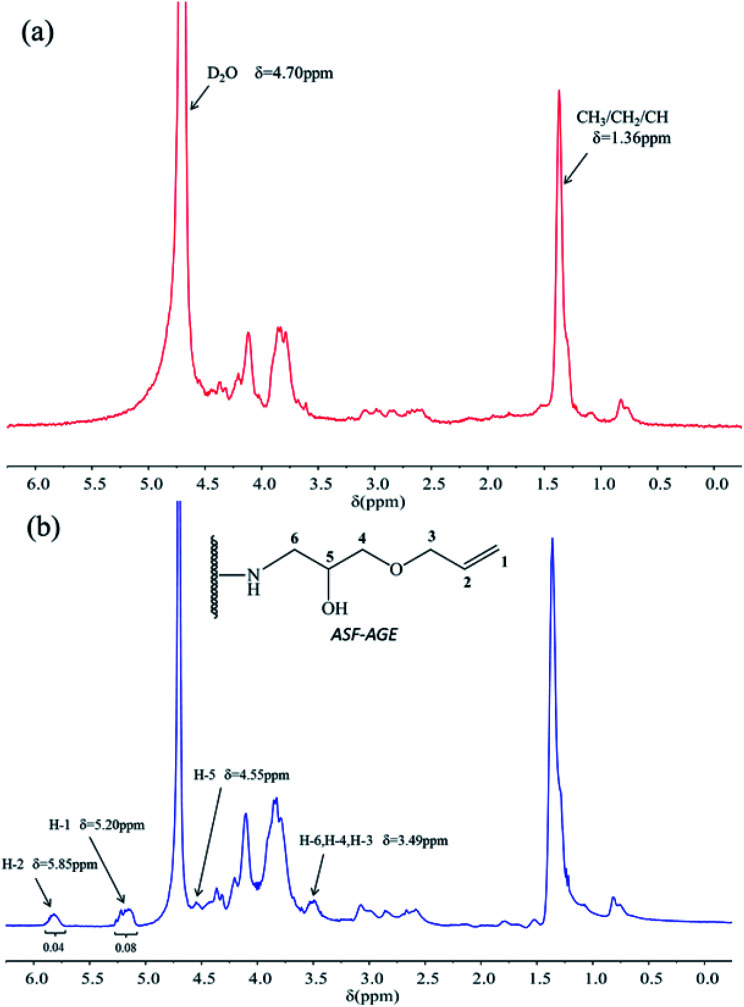
^1^H NMR spectra of (a) ASF and (b) ASF-AGE.

For the ^1^H NMR spectra of ASF and ASF-AGE were shown in Fig. 3, the peak of ASF-AGE (Fig. 3(b)) appearing at 5.20 ppm (H-1) and 5.85 ppm (H-2) which was mainly attributed to the characteristic peaks of protons (^1^H) resonances on the vinyl, and the peaks integral ratio of H-1 and H-2 was 2 : 1. The chemical shift at 4.55 ppm in Fig. 3(b) was attributed to the characteristic peak of ring-opening epoxy group (H-5), and the peak of 3.49 ppm was corresponding to the methylene protons (^1^H) resonances. While there was no corresponding chemical shift characteristic peaks in the spectra of ASF (Fig. 3(a)). According to the ^1^H NMR spectrum, it was found that nucleophilic substitution was occurred with ASF amino group and AGE epoxy group, and reactive allyl C=C were obtained on ASF.

**Fig. 4:**
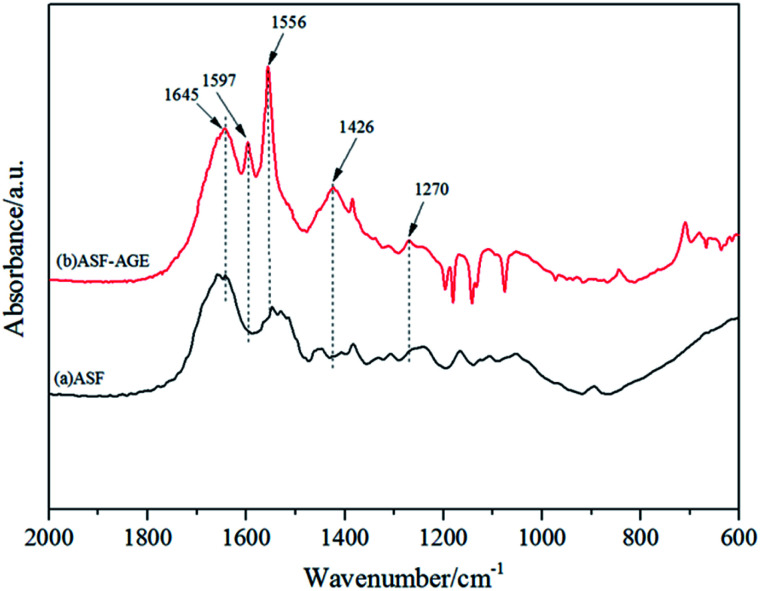
FTIR spectra of ASF and ASF-AGE.

The structures of ASF and ASF-AGE were analyzed using FTIR respectively, and the spectrum curves were depicted in Fig. 4. It was shown that the characteristic absorption peaks of ASF-AGE at 1579 cm^−1^ and 1426 cm^−1^, which was ascribed to C=C stretching, and C=C–H bending. The infrared spectral region within 1700–1500 cm^−1^ is assigned to absorption by the peptide backbones of amide I (1700–1600 cm^−1^) and amide II (1600–1500 cm^−1^). The absorption bands at 1645, 1556, and 1270 cm^−1^ were due to the peaks of amides I, II, and III, respectively.^27^ It was confirmed that the formation of allyl silk fibroin during the nucleophilic substitution.

### 3.2 Preparation of p(ASF-*a*-NIPAAm) hydrogels

PNIPAAm and silk fibroin hydrogels have been extensively studied for drug carriers, tissue engineering and gene delivery.^28–31^ ASF hydrogel can be easily prepared from ASF solution by inducing β-sheet structure formation to form physical crosslinking.^32^ PNIPAAm hydrogel can be taken shape in the presence of crosslinking agent. Cross-linked PNIPAm exhibits drastic swelling/de-swelling transition at its LCST.^33^ However, conventional PNIPAAm hydrogel and ASF-based hydrogel have been notorious for the poor mechanical properties, hinder their applications for the past decades. Herein, p(ASF-AGE-NIPAAm) hydrogels were fabricated by solution free-radical batch polymerization without any crosslinking agent.

**Fig. 5:**
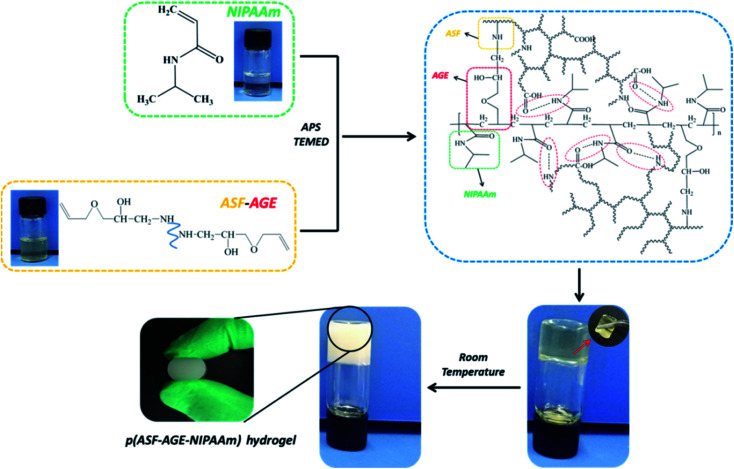
Preparation of p(ASF-AGE-NIPAAm) hydrogel.

**Fig. 6:**
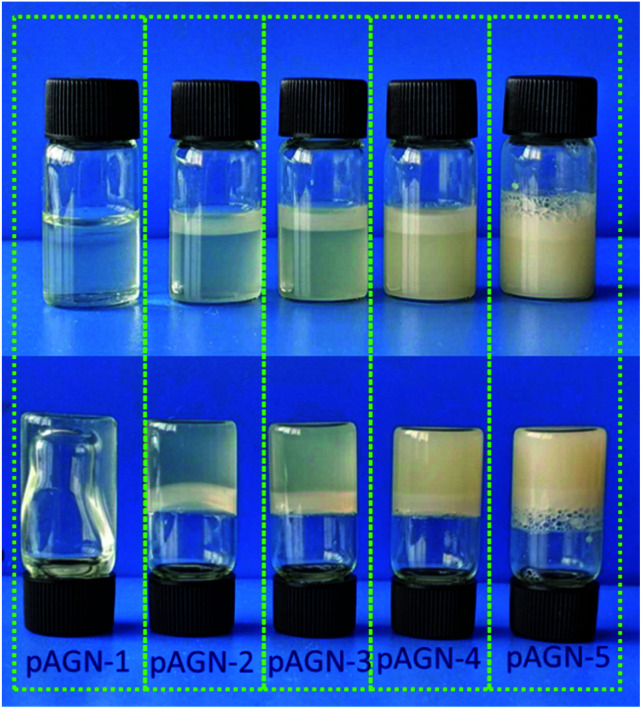
Image of p(ASF-AGE-NIPAAm) hydrogels with different mass ratios.

The preparation of p(ASF-AGE-NIPAAm) hydrogel was shown in Fig. 5. A mount of SO_4_^−^ radicals were decomposed by APS under the catalysis of TEMED which attacked the vinyl C=C of ASF-AGE and NIPAAm, addition polymerization were occurred. It was covalently connected with the allyl C=C in ASF-AGE together with the formation of PNIPAAm polymer long chain. Moreover, the ASF peptide chains were bridged the PNIPAAm by the formation of hydrogen bond. Ultimately, stably physical cross-linked hydrogels were formed by the β-sheets structure formation of ASF. The addition of more ASF-AGE resulted in the formation of hydrogels from the solutions as shown in Fig. 6. It was found that the gelation process of p(ASF-AGE-NIPAAm) hydrogels was formed from top to bottom. It was the typical gelation process of silk fibroin. It can be seen that the faint yellow solutions become non-transparent and non-flowed gels, and with the increase of ASF-AGE, the excellent shape of hydrogels was formed in Fig. 6. As the mass ratio of ASF-AGE/NIPAAm became 1 : 1.5 (pAGN-4), a completely opaque but non-fluidic hydrogel was obtained. As the ASF-AGE content was further increased, typical hydrogel was formed and a part of silk fibroin was separated out from hydrogel. The sol–gel transition of silk fibroin arises from a combination of inter and intramolecular interactions including hydrophobic interactions and hydrogen bonds leading to β-sheets formation, resulting in physical cross-links. Fig. 6 showed that increased concentration of silk fibroin provides the enhanced chain interactions and thus the hydrogel was more completed. These results suggested that stabilized hydrogel can be taken shape by ASF-AGE and NIPAAm copolymerization. As the further increased of ASF-AGE content, the hydrogel was more densely connected or cross-linked, leading to redundant silk fibroin separate out and poor compatibility.

### 3.3 Thermoresponsive of p(ASF-AGE-NIPAAm) hydrogels

**Fig. 7:**
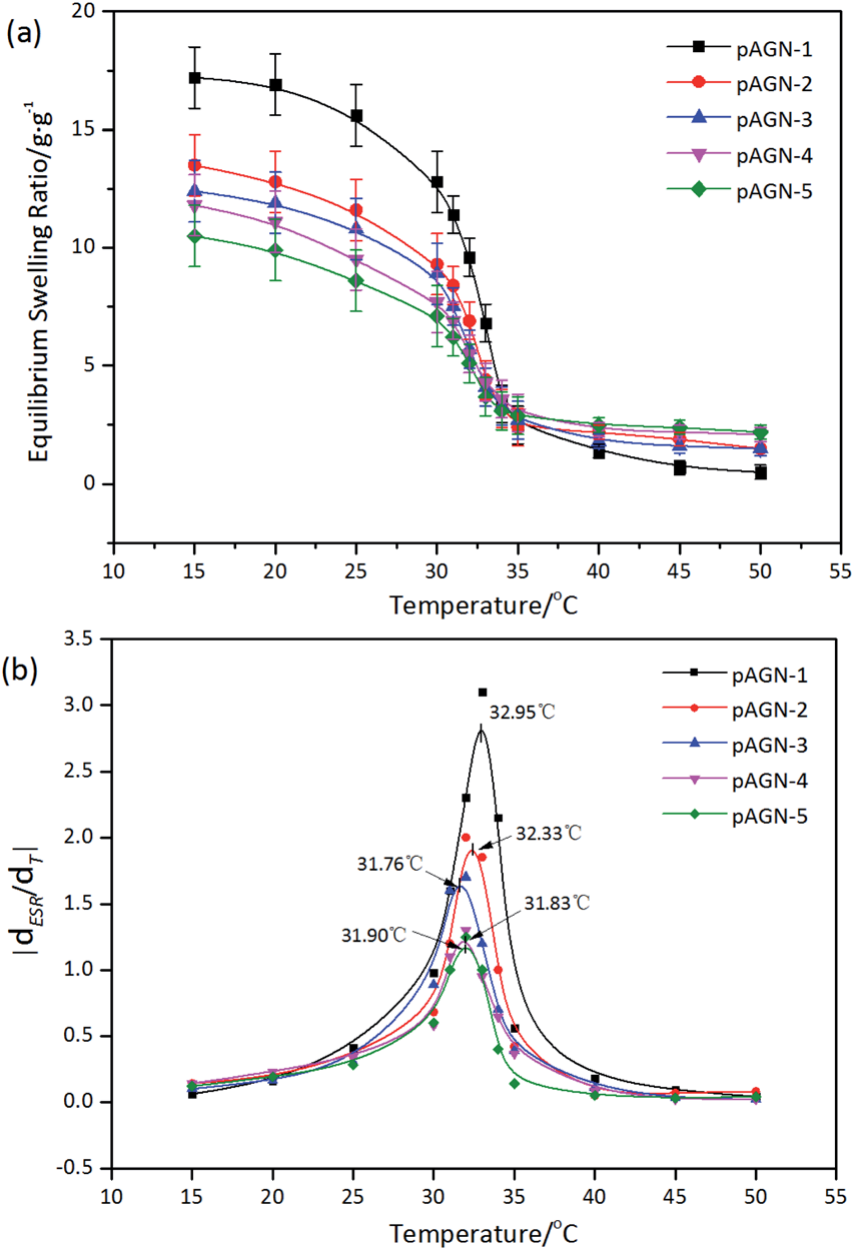
(a) EMC and (b) LCST for prepared hydrogel with different mass ratio of ASF-AGE to NIPAAm.

The temperature dependence of equilibrium moisture content (EMC) was shown in Fig. 7(a). With the increasing temperature, the hydrogels gradually shrank to equilibrium. It was shown that each hydrogel samples have a similar classical thermoresponsive profile. It is well-known that PNIPAAm hydrogels have a LCST of around 32 °C, a very useful temperature for biomedical applications since it is close to the body temperature (37 °C). PNIPAAm is one typical polymer which shrinks in high temperature. A polymer solution below the LCST is clear, homogeneous solution while a polymer solution above the LCST appears cloudy.^34^ Hydrogen bonds between water and hydrophilic groups make the hydrogels swelling when the temperature is below the LCST. Once the external temperature increases above the LCST, the hydrophobic interaction among hydrophobic groups dominates, resulting in a phase separation and shrinkage of hydrogels.^35^ The EMC of each hydrogel decreased dramatically towards the LCST and had the sharpest decrease around 32 °C. For p(ASF-AGE-NIPAAm) hydrogels, the LCST was about 32 °C in Fig. 7(b). Each hydrogel was shown different EMC level with different mass ratio of ASF-AGE to NIPAAm under LCST. With the increased of ASF-AGE content the LCST of each hydrogels were 32.95 °C, 32.33 °C, 31.76 °C, 31.83 °C, 31.90 °C (*p* < 0.05). This behavior could be explained by the enhancement of physical crosslinking through introducing plenty ASF. This result suggested that p(ASF-AGE-NIPAAm) hydrogels had the same thermoresponsive with PNIPAAm hydrogel. The EMC of the hydrogels under LCST can be well controlled by changing the ASF-AGE content within the hydrogels.

### 3.4 Degradation behavior of p(ASF-AGE-NIPAAm) hydrogels

**Fig. 8:**
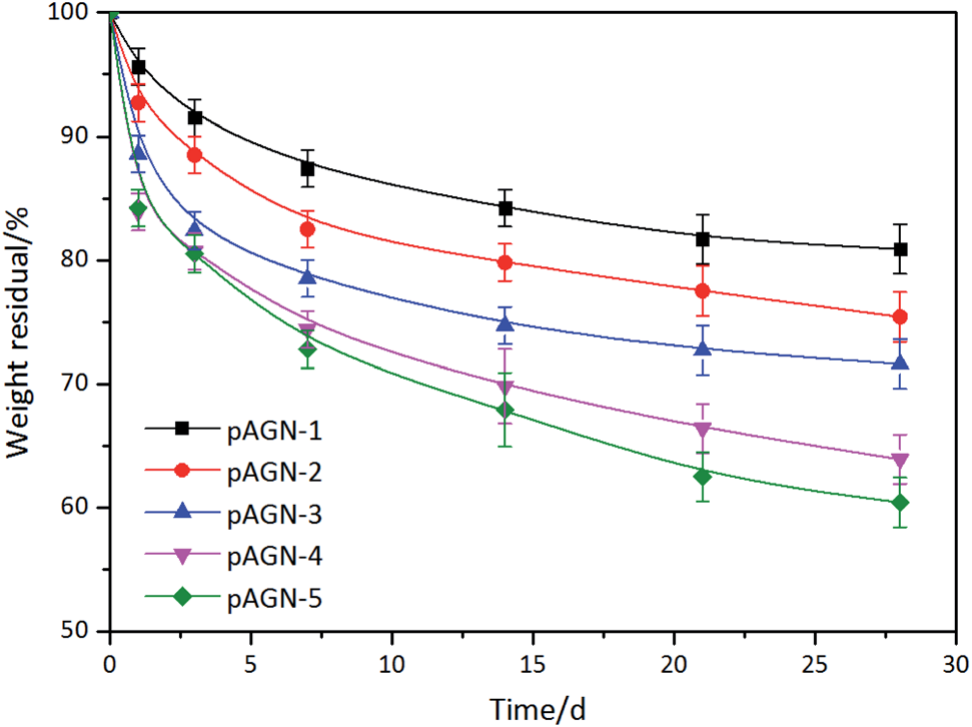
Enzymatic degradation behavior of the p(ASF-AGE-NIPAAm) hydrogels.

Silk fibroin can be degraded by proteinase *in vitro* and *in vivo*, and the degradation products of silk based materials are soluble peptides and free amino acids, which are easily metabolized and absorbed by the body.^36,37^ In this study, enzymatic degradation behavior of p(ASF-AGEA-NIPAAm) hydrogels were investigated. The changes of weight loss of hydrogels over the degradation time were calculated. As shown in Fig. 8, the weight loss of hydrogel followed a clear trend after exposure to protease XIV solution. With the extension of incubated time in protease XIV solution, the weight loss of hydrogels was increased. After 28 days, the weight loss of hydrogels was reached to 19.1–39.6% (*p* < 0.05). It was shown that increased weight loss with the increase of ASF-AGE amount, and the hydrogel with more ASF-AGE amount showed a more rapid degradation. This result suggested small molecular peptides were released from hydrogel under the degradation of protease XIV, and the ASF-AGE component in hydrogel was significantly enhanced the enzymatic degradability. It has been reported that the molecular weight and chemical crosslinking of regenerated silk fibroin affect the degradation rate of silk based biomaterials.^38^ Hence, the degradation mechanism of p(ASF-AGE-NIPAAm) hydrogel was mainly the degradation of silk fibroin. The degradation properties of silk fibroin based materials are correlated with the β-sheet content. In our previous research, it was found that the silk I structure was increase with the increase of ASF.^39^ In this work, the formation of p(ASF-AGE-NIPAAm) hydrogels were mainly physical cross-linked structure without any chemical cross-linked components, and the aggregated structure of hydrogels was mainly existed in the form of amorphous and lower metastable β-sheet physical structure. The amorphous regions and metastable silk I structure were preferentially degraded in regenerated silk fibroin materials, the lower β-sheet content contributed to a less crystalline structure that allowed easier enzyme degradation.^40,41^ Therefore, the p(ASF-AGE-NIPAAm) hydrogels are more easily degraded compared with other chemically cross-lined hydrogel. *In vitro* degradation results indicated that p(ASF-AGE-NIPAAm) hydrogels can be degraded in protease XIV solution, and the ASF-AGE component significantly promoted the degradability of the hydrogels.

### 3.5 Cell adhesion and growth on p(ASF-a-NIPAAm) hydrogels

**Fig. 9:**
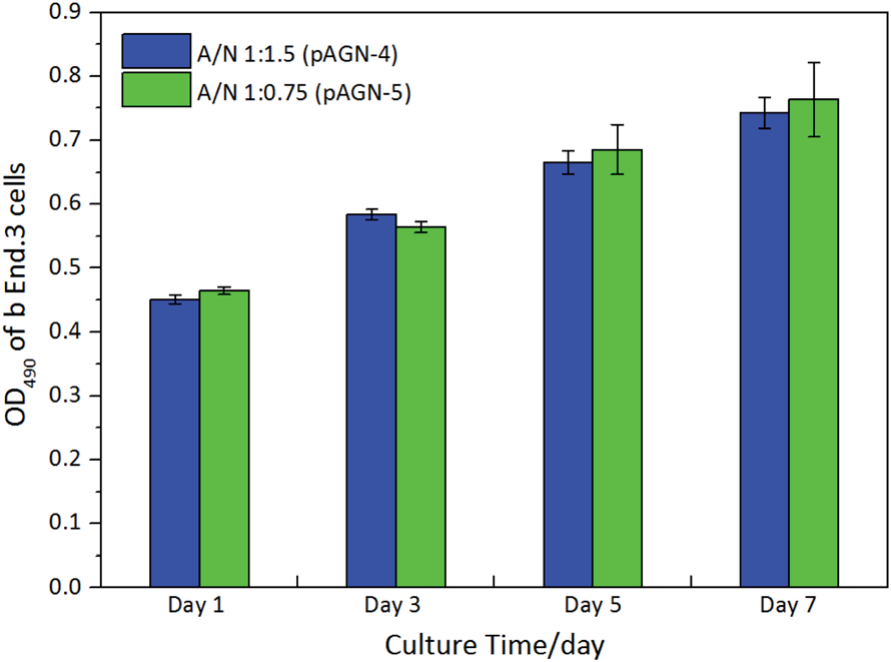
Cell viability measured by MTT.

In order to evaluate whether the hydrogels can support cell adhesion and growth, brain microvascular endothelial cells (b End.3) were cultured with the p(ASF-AGE-NIPAAm) hydrogel samples (pAGN-4, pAGN-5). The yellow MTT is reduced to a purple formazan dye by mitochondrial dehydrogenase in living cells, and can be used to assess cell viability.^42^ After cultured, the cells were stained with DAPI (blue) for the nucleus. The OD values of purple formazan at 490 nm are proportional to the number density of adhered cells. As shown in Fig. 9, the OD values at 490 nm were increased over time. At an ASF-AGE and NIPAAm mass ratio of 1 : 0.75 (pAGN-5), more cells adhered on the samples than at a ratio of 1 : 1.5 (pAGN-4).

**Fig. 10:**
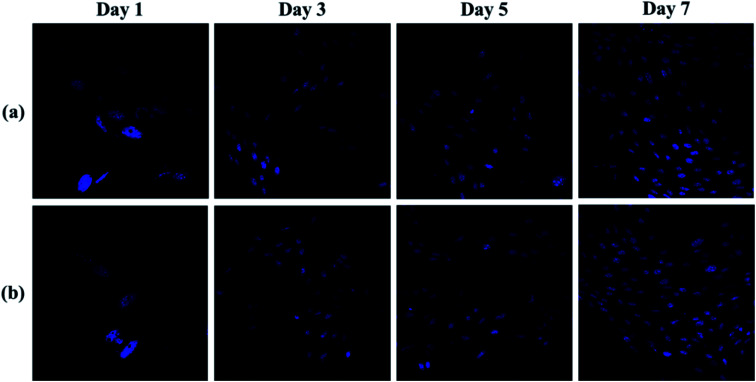
CLSM images of b End.3 cells labeled by DAPI: hydrogel samples of (a) pAGN-4 and (b) pAGN-5.

Fig. 10 was shown the CLSM images of the cells cultured on the hydrogels (pAGN-4, pAGN-5) substrate for 1, 3, 5 and 7 days. As shown in Fig. 10, few cells adhered on the hydrogel substrate at day 1. The number of cells was increased by several times at day 3. The number density of oval cells was significantly increased at day 5 and 7. From day 1 to day 7, it is obvious that the cell number and growth was higher on substrates with the extension of time in parallel comparison. It was indicated that the presence of ASF-AGE was beneficial for cell growth and adhesion on the ASF-AGE based hydrogels (pAGN-4 or pAGN-5). In vertical comparison, it can be seen the cell number and growth of pAGN-5 was a little higher than pAGN-4 from day 3 to day 7, the cell number density and growth was higher on substrates with a higher ASF-AGE content. Furthermore, MTT assay was also confirmed this result. The cell culture results suggested that the presence of ASF-AGE was beneficial for cell adhesion and growth on the p(ASF-AGE-NIPAAm) hydrogels and the cell adhesion density was more obvious at abundant of ASF-AGE.

**Fig. 11:**
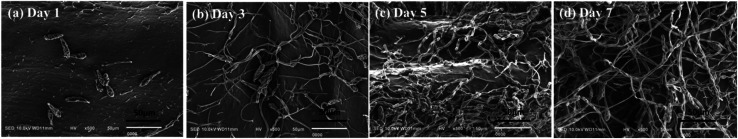
SEM photographs of the b End.3 cells seeded on hydrogel surface of pAGN-5: (a)–(d) incubation for 1, 3, 5, 7 days respectively.

Fig. 11 was shown the SEM images of b End.3 cells grown on pAGN-5 hydrogel sample. A good vascular graft could promote cell–scaffold interactions and maintain cell viability during the culture period. It was shown that the b End.3 cells adhered well 1 day after seeding in Fig. 11(a). The cells had proliferated and adopted spindle morphology, spread and started to form interconnections after 5 days in Fig. 11(c). After incubation for 7 days, cells completely covered the internal surface and had formed a more extensive interconnected network (Fig. 11(d)).

*A. pernyi* silk fibroin matrices have appealing features for tissue engineering and regenerative applications including cell culture substrates, bone regeneration bioactive controlled release carriers, and gene delivery systems.^43–45^*A. pernyi* silk fibroin is rich in arginyl-glycyl-aspartic acid (RGD) peptides, which are binding sites for cell integrin receptors. They mediate the interactions between mammalian cells and extracellular matrices.^13^ The cell culture results in this work also demonstrated that the presence of *A. pernyi* silk fibroin was beneficial for cell adhesion and growth on the p(ASF-AGE-NIPAAm) hydrogels. With the combination of thermo-sensitivity and degradability, these *A. pernyi* silk fibroin based thermo-sensitivity hydrogels will be found potential applications in delivery device of cells or envisioned for restoring biomedical and biochemical functions of different tissues.

## 4. Conclusions

Allyl silk fibroin has been prepared by nucleophilic substitution reaction of ASF and AGE. And then the p(ASF-AGE-NIPAAm) thermoresponsive hydrogels have been prepared by copolymerization of ASF-AGE and NIPAAm through physical cross-linking by the β-sheets structure formation of ASF. These obtained hydrogels have obvious thermoresponsive and the LCST of the hydrogels are about 32 °C. Furthermore, these hydrogels can be degraded in protease XIV solution, and the ASF-AGE component significantly promoted the degradability of the hydrogels. By culturing brain microvascular endothelial cells *in vitro*, the hydrogels support cell adhesion and growth well. These degradable and thermoresponsive hydrogels will have potential applications for cells delivery device and tissue scaffold.

## Conflicts of interest

There are no conflicts to declare.

## Supplementary Material
